# Gradual rewarming with a hemoglobin-based oxygen carrier improves viability of donation after circulatory death in rat livers

**DOI:** 10.3389/frtra.2024.1353124

**Published:** 2024-07-01

**Authors:** Paria Mahboub, Mohamed Aburawi, O. Sila Ozgur, Casie Pendexter, Stephanie Cronin, Florence Min Lin, Rohil Jain, Murat N. Karabacak, Negin Karimian, Shannon N. Tessier, James F. Markmann, Heidi Yeh, Korkut Uygun

**Affiliations:** ^1^ Department of Surgery, University Medical Center Groningen, Groningen, Netherlands; ^2^ Center for Engineering in Medicine and Surgery, Massachusetts General Hospital and Harvard Medical School, Boston, MA, United States; ^3^ Department of Research, Shriners Hospitals for Children, Boston, MA, United States; ^4^ Transplant Center, Department of Surgery, Massachusetts General Hospital and Harvard Medical School, Boston, MA, United States

**Keywords:** cold storage, gradual rewarming, HBOC, machine perfusion, donation after cardiac death (DCD)

## Abstract

**Background:**

Donation after circulatory death (DCD) grafts are vital for increasing available donor organs. Gradual rewarming during machine perfusion has proven effective in mitigating reperfusion injury and enhancing graft quality. Limited data exist on artificial oxygen carriers as an effective solution to meet the increasing metabolic demand with temperature changes. The aim of the present study was to assess the efficacy and safety of utilizing a hemoglobin-based oxygen carrier (HBOC) during the gradual rewarming of DCD rat livers.

**Methods:**

Liver grafts were procured after 30 min of warm ischemia. The effect of 90 min of oxygenated rewarming perfusion from ice cold temperatures (4 °C) to 37 °C with HBOC after cold storage was evaluated and the results were compared with cold storage alone. Reperfusion at 37 °C was performed to assess the post-preservation recovery.

**Results:**

Gradual rewarming with HBOC significantly enhanced recovery, demonstrated by markedly lower lactate levels and reduced vascular resistance compared to cold-stored liver grafts. Increased bile production in the HBOC group was noted, indicating improved liver function and bile synthesis capacity. Histological examination showed reduced cellular damage and better tissue preservation in the HBOC-treated livers compared to those subjected to cold storage alone.

**Conclusion:**

This study suggests the safety of using HBOC during rewarming perfusion of rat livers as no harmful effect was detected. Furthermore, the viability assessment indicated improvement in graft function.

## Introduction

Donation after circulatory death (DCD) grafts are a major resource when expanding the organ pool (1, 2). However, DCD grafts are associated with post-transplant challenges, such as primary non-function, ischemia reperfusion injury, and biliary complications (3–5). Therefore, the improvement of DCD graft quality before implantation is an essential step toward improving the outcome after transplant.

After the procurement of grafts, donor livers undergo a period of cold storage (CS), where they are kept on ice while being transported. During this period, hypoxic conditions lead to adenosine triphosphate (ATP) depletion and build-up of toxic metabolites, resulting in progressive organ deterioration (6). The combination of warm and cold ischemic injury is suggested to increase ischemia reperfusion injury, which explains the inferior quality of DCD grafts after transplantation (4). Machine perfusion (MP) is a new preservation method currently in clinical trials across the world and offers the likelihood of treating these DCD livers (7–11). Different MP protocols from hypothermic to sub-normothermic and normothermic have been studied before and the results have shown that machine perfusion is beneficial in reducing reperfusion injury (12–14). Recent clinical trials showed significant increase in DCD liver graft use after MP compared to CS, with notable reduction in early allograft dysfunction (EAD) and ischemic biliary complications (IBC) (9, 11). Among different perfusion protocols, normothermic machine perfusion with red blood cells (RBCs) is one of the most common methods in clinical trials as it offers viability assessment during the graft preservation period (8, 10, 11, 15).

Gradual rewarming during MP from hypothermia to normothermia has been described as a successful preservation method for eliminating reperfusion injury (16, 17). Meeting the high demand of oxygen with increasing temperatures in gradual rewarming requires the use of an oxygen carrier. In this study, the gradual rewarming with the supplementation of a hemoglobin-based oxygen carrier (HBOC) as the oxygen carrier was designed to assess the feasibility and safety of using an artificial oxygen carrier. The liver function was compared with CS alone as the baseline.

## Material and methods

### Experimental animals and liver procurement

Male Lewis rats weighing 290–350 g were used in this study. Animals received care according to the National Research Council guidelines on animal experiments. The study protocol was approved by the Institutional Animal Care and Use Committee (IACUC) at Massachusetts General Hospital. Inhalation anesthesia with 2%–3% isoflurane and oxygen was administered before and during the procurement. In anesthetized rats, anticoagulation was achieved by injecting 500 U of heparin in 1 ml of saline into the penile vein. The DCD model procedure and *in situ* warm ischemic time (30 min) have been described previously (18). During this time, the bile duct was cannulated, the portal vein was cannulated using an 18-gauge intravenous catheter, and at the end of warm ischemia period, the liver was flushed through the portal vein with 10 ml 0.9% NaCl at room temperature followed by 30 ml University of Wisconsin preservation solution (UW) at 4 °C. The liver was removed and stored in cold UW (4 °C) during CS preservation.

### Experimental design

A total of 10 rat livers were divided into two experimental groups after DCD procurement (*n* = 5 per group). In the rewarming with HBOC group (rewarm&HBOC), after 270 min CS in UW media, the liver grafts underwent gradual rewarming perfusion from 8 °C to 37 °C for 90 min. Later, the grafts were flushed with 10 ml of cold saline and stored in a Petri dish covered with wet gauze at room temperature for 30 min to mimic the surgical implantation period. Subsequently, the grafts were reperfused at 37 °C for 120 min, with the protocol detailed below. In the CS group, the grafts were kept in ice cold UW for 360 min to align the total storage time of both groups. Subsequently, a 30-min storage period at room temperature was implemented to simulate implantation conditions, followed by reperfusion at 37 °C for 120 min, as in the rewarming group.

### Machine perfusion

**Figure 1:**
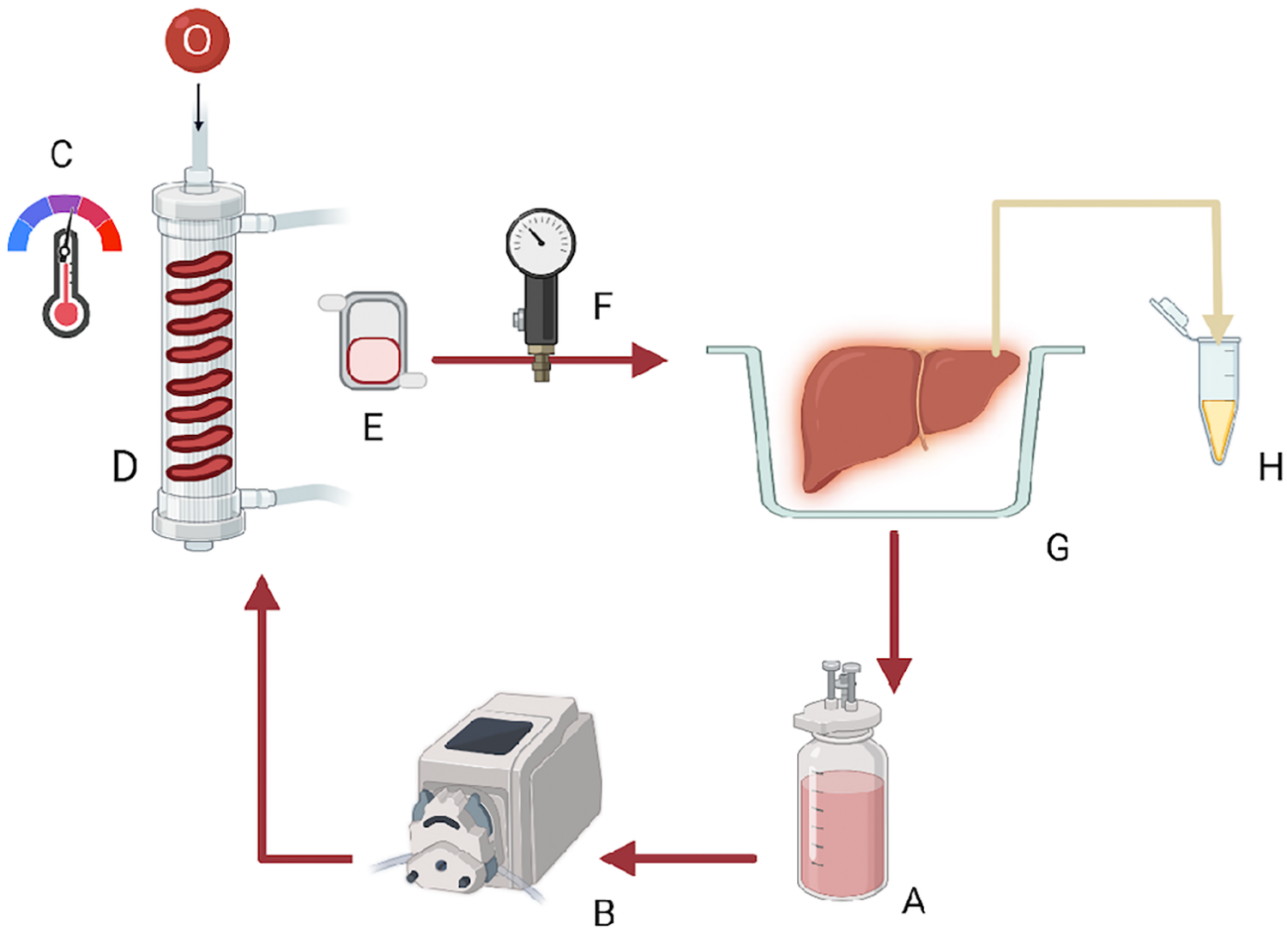
Graphic representation of the rodent liver perfusion system: (**A**) solution reservoir, (**B**) roller pump, (**C,D**) oxygenator containing silicon tubing and providing Carbogen and a heat exchanger with a thermoregulator, (**E**) bubble trap, (**F**) pressure probe, (**G**) organ chamber, and (**H**) bile Eppendorf.

The perfusion device is a flow-controlled system for rodent organ perfusion (Figure 1) (19). In the rewarming group, the temperature of the perfusion solution was set at 8 °C at the beginning of rewarming, the minimum allowed by the equipment, and was gradually increased to 37 °C through 60 min. The temperature was kept stable at 37 °C for the final 30 min.

### Viability assessment: normothermic reperfusion

To mimic the transplant process, a 30-min room temperature period was chosen to reflect the implantation in the recipient as described above followed by a 120 min normothermic perfusion duration, which was considered a simulated early post-transplantation period. In both the experiment and control groups, the perfusion temperature was set at 37 °C and perfusion was continued for a total of 120 min. The flow rate during this period was initially set at 8.0 ml/min and was adjusted according to the portal venous pressure, which was regulated at 50–140 mM H_2_O.

### Perfusion solution

The perfusion solution consisted of Williams Medium E (Sigma-Aldrich, St. Louis, MO, USA) supplemented with insulin (2 U/L Humulin; Eli Lilly & Co., Indianapolis, IN, USA), L-glutamine (0.292 g/L; Gibco/Invitrogen, Waltham, MA, USA), heparin (1,000 U/L APP Pharmaceuticals, Schaumberg, IL, USA), Albumin 15% (Sigma-Aldrich), and 25% HBOC-201 v/v (provided by Hemoglobin Oxygen Therapeutics, Souderton, PA, USA). The total volume of perfusate was 300 ml for both the rewarming and reperfusion phases. Different HBOC concentrations were tested, and the final concentration was determined based on ensuring detectable hemoglobin in the perfusate, as well as a detectable change in the dissolved oxygen in the media. During the rewarming and reperfusion experiments, the solution was oxygenated using Carbogen, a mixture of 95% O_2_ and 5% CO_2_. Note that the same formula was used in both the gradual rewarming group and the reperfusion phase. In the rewarming group, during the 30-min simulated anastomosis time, the perfusion device was flushed clean and the perfusion solution was renewed. Bicarbonate supplementation is adjusted based on perfusate pH levels during rewarming but not during reperfusion to facilitate the clear monitoring of pH differences in both groups.

### Perfusion measurements and injury

#### Rewarming

During rewarming, temperature, flow, and pressure were recorded at 30-min intervals and subsequently resistance was calculated. pH, bicarbonate, lactate, pO_2_, and pCO_2_ levels in the perfusate samples were analyzed using an I-Stat analyzer (Abbott, Chicago, IL, USA) every 30 min, and pH was corrected by adding 8.4% NaHCO_3_^−^. Bile production was observed and recorded at the end of the 90-min rewarming.

#### Reperfusion

The same perfusion parameters noted in the rewarming procedure in addition to glucose were measured in both groups during 120 min of reperfusion.

### Liver injury

Alanine aminotransferase (ALT) was measured in the perfusate samples using an ELISA kit (MBS041480 MyBioSource, Inc., San Diego, CA, USA) during reperfusion in both rewarming and CS groups.

### Oxygen consumption and ATP measurement

#### Reperfusion

Oxygen consumption during reperfusion was calculated in both groups using the following formula: $\left([\{\mathrm{Ap}O_{2}-\mathrm{Vp}O_{2}\}\times K/760]\times \mathrm{total}\;\mathrm{flow})+[\{\mathrm{As}O_{2}-\mathrm{Vs}O_{2}\right\}$ ×(Hb×c×0.0001]×flow)/Liverweight×100. In which pO_2_ was measured in mmHg, sO_2_ in %, Hb in g/dl, portal vein flow in ml/min, and liver weight in g. c was the oxygen binding capacity of HBOC (1.26) and K was a constant (0.0225).

The tissue samples for ATP measurement were only taken at the end of reperfusion (*t* = 120 min) to prevent inducing injury to the liver grafts during rewarming and reperfusion. The method of extraction and measurement has been described previously (20, 21).

### Bile production and cholangiocyte function

#### Rewarming

The produced bile was collected in Eppendorf tubes and measured in ml at the end of the 90-min rewarming period.

#### Reperfusion

Bile production was recorded, and biliary epithelial cell function was assessed by measuring pH and bicarbonate concentration in bile (22). For this purpose, bile samples were collected under mineral oil and were analyzed immediately using the I-Stat analyzer.

### Histological evaluation

#### Reperfusion

Biopsies were obtained from the liver parenchyma at the end of reperfusion phase and were stored in 10% formalin for the histological evaluation. Paraffin-embedded slides of liver biopsies were prepared for hematoxylin and eosin (H&E).

### Statistical analysis

Continuous data were presented as the median and interquartile range (IQR). The Mann–Whitney *U*-test was used to compare groups. A *p*-value <0.05 was considered significant. Analyses were performed using SPSS version 22.0 for Windows (IBM Corp., Armonk, NY, USA).

## Results

### Perfusion profile during gradual rewarming

**Figure 2:**
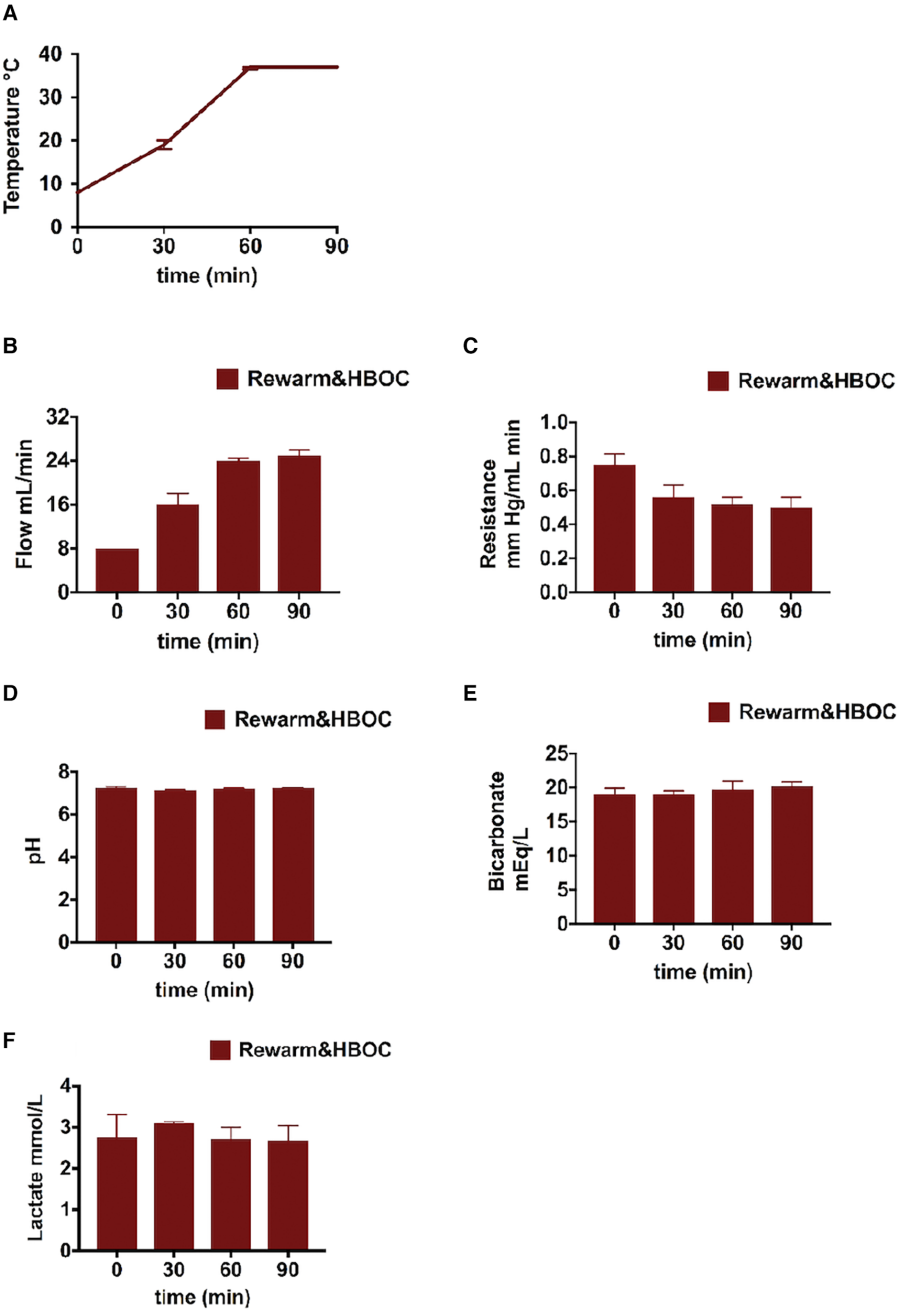
Liver profile during the 90-min gradual rewarming phase of the rewarm&HBOC group after CS: (**A**) temperature and (**B,C**) flow was increased and portal resistance slightly reduced during gradual rewarming. (**D**) pH normalized by the end of the gradual rewarming procedure. (**E**) Bicarbonate levels in perfusate. (**F**) Decrease in lactate levels.

Temperature profile during rewarming is shown in Figure 2A. Portal vein flow was increased and resistance decreased and stabilized toward the end of rewarming perfusion (Figures 2B,C). Regarding other perfusion parameters, the bicarbonate level was slightly improved with the pH within the physiological range in all the liver grafts (Figures 2D,E). The lactate level was constant through the rewarming perfusion in all the liver grafts (Figure 2F). We also observed bile production (0.45–1.25 ml over 90 min) during rewarming, which is an indicator of liver function.

### Comparison of gradual rewarming and cold storage during reperfusion

**Figure 3:**
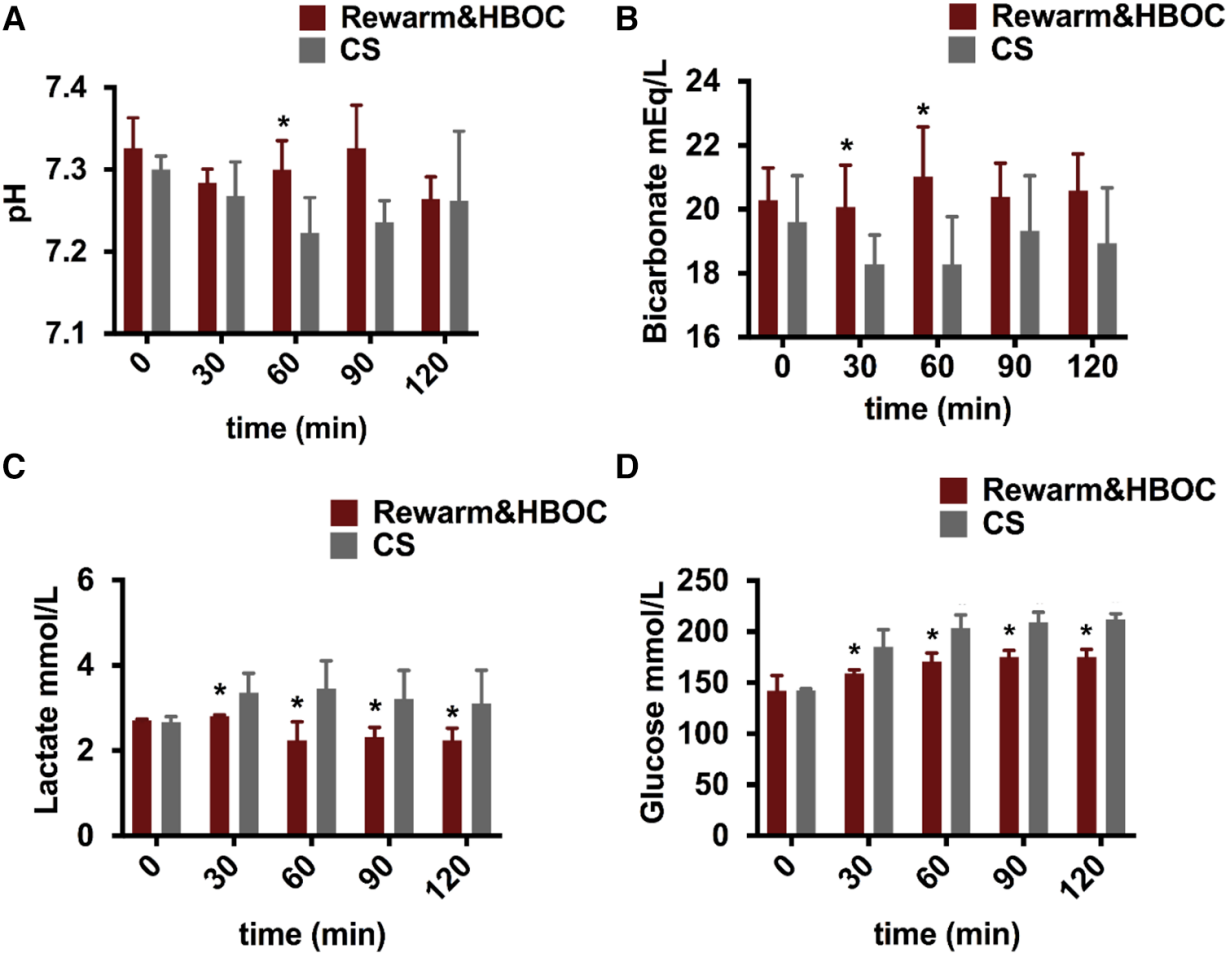
Livers undergoing gradual rewarming showed improved function and recovery compared to CS controls during the 2-h reperfusion with HBOC. (**A**) pH was better in the rewarm&HBOC group compared to the CS group, with a significant difference at *t* = 60 (*p* = 0.016). (**B**) Bicarbonate levels were better in the rewarm&HBOC group with a significant difference at *t* = 30 (*p* = 0.05) and *t* = 60 (*p* = 0.032). (**C**) In contrast to the CS group, the lactate level was significantly lower in the rewarm&HBOC group between *t* = 30 and *t* = 120 (*p* ≤ 0.05). (**D**) Glucose concentration remained significantly lower in the rewarm&HBOC group compared to the CS group between *t* = 30 and *t* = 120 (*p* = ≤0.05). * represents individual time point significances of the experimental group.

pH was closer to the physiological range in the rewarm&HBOC group compared to the CS liver grafts during 120 min of reperfusion (Figure 3A). In the rewarm&HBOC group, the bicarbonate level was higher compared to the CS group in the first hour of perfusion (Figure 3B). Lactate concentrations displayed a slight initial increase, followed by a slight decrease in both groups, with concentration remaining lower in the rewarm&HBOC liver grafts compared to CS liver grafts throughout the rest of reperfusion procedure (between *t* = 30 and *t* = 120; *p* ≤ 0.05) (Figure 3C). The glucose concentration was measured in the perfusate samples during 120 min of reperfusion and was lower in the rewarm&HBOC from *t* = 30 until the end of reperfusion (*p* ≤ 0.05) (Figure 3D).

### Liver injury

**Figure 4:**
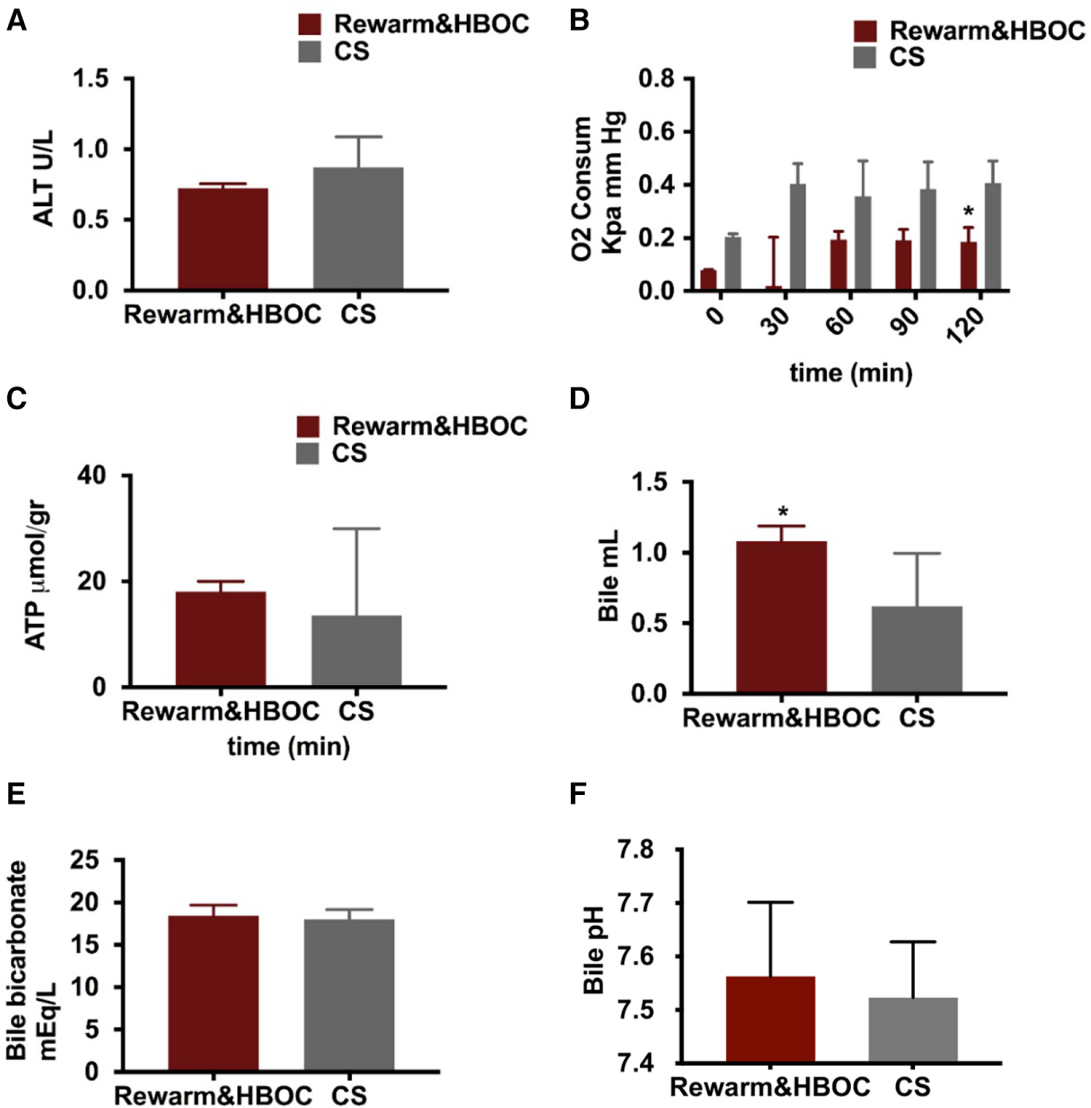
Graphical presentation of oxygen concentration, ATP level, and bile fluid in both the rewarming and CS groups. (**A**) There was a lower trend of ALT in the rewarm&HBOC group compared to the CS group during 120 min of reperfusion (*p* = 0.056). (**B**) Oxygen consumption remained higher in the CS group in comparison with the rewarming group, and this difference was significant at *t* = 90 (*p* = 0.024). (**C**) There was no significant difference in ATP levels between both groups (*p* = 0.55). (**D**) The total volume of bile production measured at the end of 120 min of reperfusion was meaningfully higher in the rewarm&HBOC group compared to the CS group. (**E**) There was no difference in the level of bicarbonate and (**F**) bile pH in bile samples of the rewarm&HBOC and CS groups during 120 min of reperfusion. * represents individual time point significances.

ALT, an indicator of hepatic injury, was measured in the perfusate samples during reperfusion. ALT in the rewarm&HBOC group was somewhat lower compared to that in the CS group, although it did not reach significance, with *p* = 0.056 (Figure 4A).

### Oxygen consumption and ATP

Oxygen consumption was measured during 120 min of reperfusion and was slightly higher in the CS group in comparison to that in the rewarm&HBOC group, with a significant difference at *t* = 120 (*p* = 0.01) (Figure 4B). After 120 min of reperfusion, no statistical differences were found in the ATP production between the rewarm&HBOC and CS groups (*p* = 0.55) (Figure 4C), although this appeared to be more a result of the high variability in the CS livers and values trended higher with less variability in the rewarm&HBOC group.

### Bile production and cholangiocyte function

Cumulative bile production and biliary cholangiocyte function were measured, the median bile production was higher in the rewarm&HBOC group in comparison with the CS group (*p* = 0.03) (Figure 4D). There was no difference in the level of bicarbonate (*p* = 0.6) (Figure 4E) and pH (*p* = 0.71) (Figure 4F) and in the bile samples between the rewarm&HBOC and CS groups.

### Vascular parameters and logical evaluation

**Figure 5:**
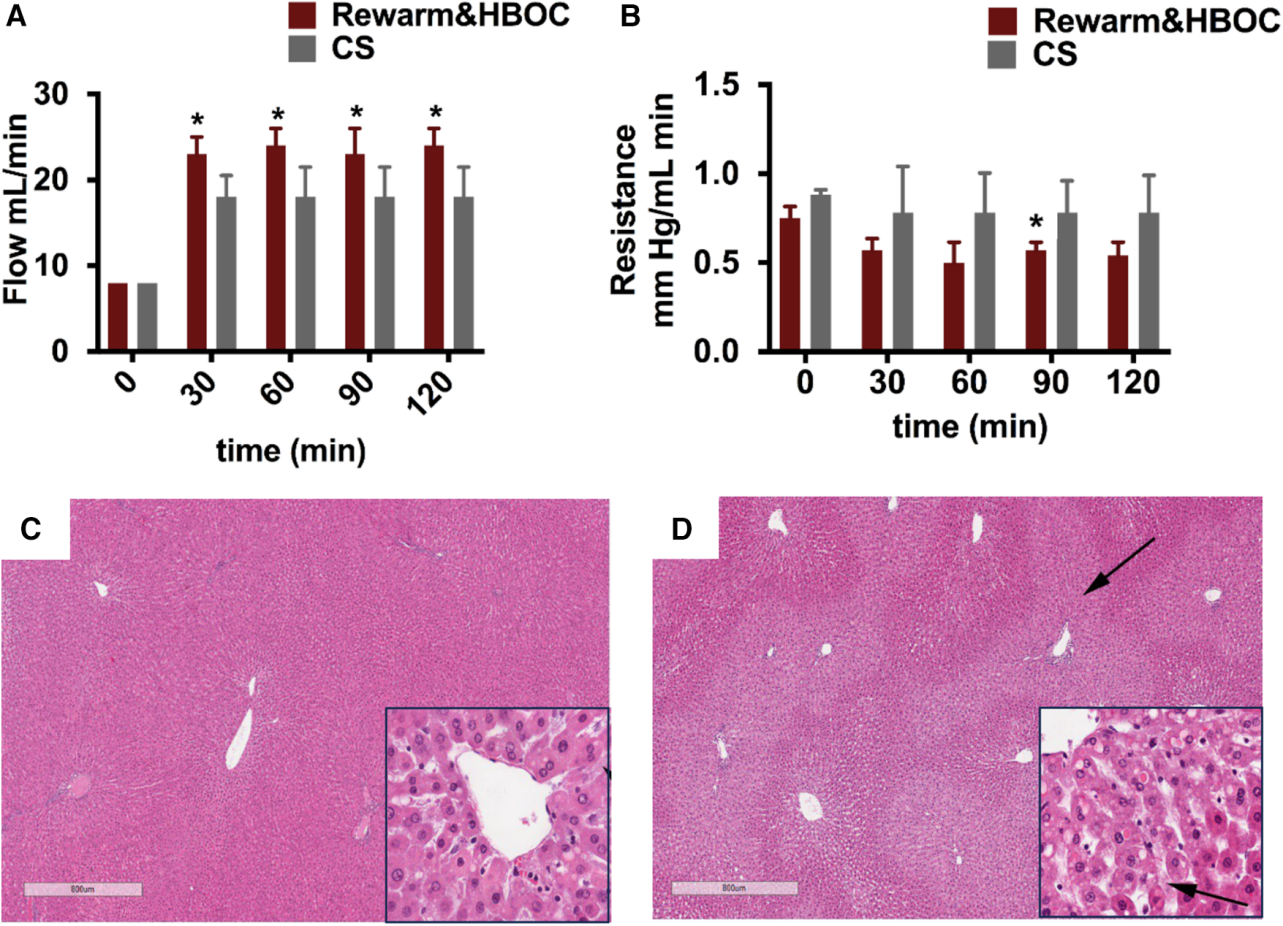
Vascular injury parameters and H&E staining from the rewarming and CS groups at the end of reperfusion. (**A**) Flow was significantly higher in the rewarm&HBOC group compared to the CS group from *t* = 30 to *t* = 120 (*p* ≤ 0.05). (**B**) Resistance was lower in the rewarm&HBOC group compared to the CS group during 120 min of reperfusion with a significant difference at *t* = 90 (*p* = 0.02). CS liver tissues (**D**) demonstrated higher venous congestion (stasis of fluid in parenchyma shown by arrows) compared to the rewarm&HBOC liver grafts (**C**).

As flow rate was increased, resistance in the portal vein gradually decreased in both the rewarming and CS groups, with lower values attained in the rewarm&HBOC group (Figures 5A,B). Overall, it was possible to obtain higher flow rates in livers in the rewarm&HBOC group consistently with pressures that were statistically the same or lower compared to those of CS controls. In parallel with the observed differences, the livers in the CS group showed more signs of venous congestion compared to those in the rewarm&HBOC group (Figures 5C,D).

## Discussion

There are two key methods used in organ preservation: traditional CS and MP. CS remains the clinical standard owing to its simplicity and low costs. However, CS can exacerbate organ deterioration, particularly in DCD grafts that have already suffered from a hypoxic period. MP is a groundbreaking approach in this field, enabling supplementation of nutrients combined with continuous oxygenation. Previous studies showed significant improvements of post-transplantation outcomes in DCD grafts after normothermic MP compared to CS (11). Although normothermic MP sustains physiological conditions and supplies vital substrates, it triggers a rapid surge in the demand for nutrients and oxygen subsequent to CS that may exacerbate reperfusion injury. The gradual and slow rewarming process, coupled with metabolic support, could potentially alleviate reperfusion injury after cold storage. A single-center clinical trial demonstrated improved early allograft function in livers subjected to rewarming after CS compared to CS alone (23). Comparing both reconditioning protocols, gradual rewarming and normothermic MP, gradual rewarming of liver grafts demonstrated superior results in terms of better energetic recovery and improved function (24). Markedly, gradual rewarming appears to mitigate rewarming injury by adapting to enhance metabolic upregulation.

Effective oxygen delivery during machine perfusion is essential for preserving organ viability and function. A major challenge with increasing temperatures during gradual rewarming is maintaining adequate oxygenation, as the oxygen content in water-based solutions decreases with rising temperatures. Therefore, an additional oxygen carrier is necessary, particularly at temperatures above 20 °C (25). Blood-based products used in machine perfusion provide effective oxygenation and have been shown to remain stable across various temperatures. The utilization of RBCs during gradual rewarming has been demonstrated by van Leeuwen et al. when compared to HBOC, revealing no significant difference between outcomes (26). Despite their efficacy, blood products come with several logistical challenges, including limited availability, high costs, and short shelf life. Moreover, blood-based perfusates may increase the risk of microvascular failure and bacterial growth (27, 28).

Artificial oxygen carriers enhance the rate of oxygenation, while avoiding these adverse events (29, 30). A number of novel oxygen carriers, such as perfluorocarbons (PFCs) and Hemarina (M101), have been developed and used in different organ preservation protocols, such as hypothermic perfusion and cold storage (31), and could be other alternatives. However, the early outcome of adding PFC in kidney machine perfusion showed instability and an adverse effect of PFC on renal function, which limits further use of this oxygen carrier in the perfusion experiment (32, 33). M101 is another novel oxygen carrier introduced recently and has a very high affinity for oxygen. Thuillier et al. have shown that adding Hemarina during cold storage to the preservation saluting improves renal function; however, there is no evidence of using M101 in liver preservation (34). HBOC has been utilized in numerous liver perfusion protocols and has previously demonstrated stability with no adverse effects at different temperatures (24, 25, 35), a crucial factor for the gradual rewarming protocol.

This study shows the feasibility of using HBOC in gradual rewarming of the liver and some end points may suggest improved graft function in rewarming using HBOC compared to the clinical standard, CS preservation. We opted for a 90-min rewarming duration, aligning with temperature stabilization within this timeframe (23), while a 120-min duration for reperfusion has been identified as optimal for *ex vivo* organ assessment (36). The outcomes of reperfusion are indicated by better flow rate and physiologically balanced perfusion pH and bicarbonate during reperfusion. Better bicarbonate concentration is a result of the liver using up the lactate; therefore, better lactate clearance results in more balanced bicarbonate and pH. The absence of an initial lactate peak in both groups is likely a result of metabolite dilution in the perfusion solution. Regardless, these findings also explain the significantly reduced lactate in the rewarm&HBOC group during reperfusion.

Increased bile production in the rewarm&HBOC group suggests improved liver function as bile production is an early indicator of liver function. Nonetheless, there was no notable difference in bile bicarbonate and bile pH levels between the groups.

The use of HBOC in patients has been reported to induce vasoconstriction and lead to systemic hypertension in some cases (37). In contrast to these earlier studies, however, no evidence of hypertension was detected in our rewarming model and we even experienced positive effects highlighted by significantly higher flow rate and lower trend of vascular resistance in the rewarm&HBOC group. This outcome is in line with the use of HBOC in sub-normothermic and normothermic liver perfusion studies in which no negative effect of HBOC on perfusion pressure and resistance was reported (30, 35). In the rewarm&HBOC group, lactate concentration declined and glucose level in the perfusate remained significantly lower, which demonstrates that the livers in this group had superior lactate and glucose metabolism and better liver function. Low ALT concentration (*p* = 0.056) in the perfusate samples in the rewarm&HBOC group may indicate lower liver parenchyma injury. Our findings are in concordance with previous rewarming studies showing improved liver transaminase levels with gradual rewarming after CS compared to CS alone (23). The histological examination showed higher liver congestion in the CS group. It has been shown in the literature that hepatic congestion could increase liver enzymes and lead to liver injury (38).

Oxygen consumption was found to be higher toward the end of reperfusion in the CS group compared to the rewarming group with no significant difference in ATP production. This finding is in line with previous results in which the investigators found higher oxygen consumption during reperfusion after prolonged CS preservation, compared to well-preserved liver grafts (39, 40). This difference in oxygen consumption was explained previously by referring to respiratory burst and oxygen debt in severely injured post-ischemic livers with no meaningful increase in ATP production (40), and our results are in concurrence.

The present study has some limitations. In future studies, the function of liver sinosoidal endothelial cells (LSECs) will be evaluated for further assessment of the endothelial structure, and research endeavors involving trials of gradual rewarming with other oxygen carriers will provide better understanding in isolated effects of HBOC. The potential dilution effect during rewarming will be further evaluated with the inclusion of an additional hypothermic machine perfusion group. Investigating reactive oxygen species (ROS) and cytokine profile will also provide insights on the oxygenation benefit of HBOC. While these studies yield informative data, transplantation procedures are crucial for understanding the practical implications. Importantly, the expected cost of incorporating an oxygen carrier into MP is a crucial consideration. To date, this remains uncertain since artificial carriers are not commercially available. However, the presence of multiple competing products in this domain increases the likelihood of reasonable pricing in the future.

In conclusion, this study shows the feasibility of gradual rewarming with the use of HBOC, and increased efficacy in the recovery of DCD liver grafts compared to CS controls in a DCD rat model.

## Data Availability

The original contributions presented in the study are included in the article/Supplementary Material, further inquiries can be directed to the corresponding author.
